# A Time-Critical Adaptive Approach for Visualizing Natural Scenes on Different Devices

**DOI:** 10.1371/journal.pone.0117586

**Published:** 2015-02-27

**Authors:** Tianyang Dong, Siyuan Liu, Jiajia Xia, Jing Fan, Ling Zhang

**Affiliations:** 1 School of Computer Science and Technology, Zhejiang University of Technology, Hangzhou, Zhejiang, China; 2 Key Laboratory of Visual Media Intelligent Processing Technology of Zhejiang Province, Hangzhou, Zhejiang, China; Xiamen University, CHINA

## Abstract

To automatically adapt to various hardware and software environments on different devices, this paper presents a time-critical adaptive approach for visualizing natural scenes. In this method, a simplified expression of a tree model is used for different devices. The best rendering scheme is intelligently selected to generate a particular scene by estimating the rendering time of trees based on their visual importance. Therefore, this approach can ensure the reality of natural scenes while maintaining a constant frame rate for their interactive display. To verify its effectiveness and flexibility, this method is applied in different devices, such as a desktop computer, laptop, iPad and smart phone. Applications show that the method proposed in this paper can not only adapt to devices with different computing abilities and system resources very well but can also achieve rather good visual realism and a constant frame rate for natural scenes.

## Introduction

With the development of the means to acquire plant data from natural scenes, such as with the use of photogrammetry and laser scanning, the accuracy of acquiring data and the speed of modeling natural scenes have improved rapidly, resulting in improvements in the details of natural scenes and increases in the complexity and data volume of scenes. However, the hardware for 3D display has not yet satisfied the increasing demand for large amounts of scene data. Even top-of-the-line hardware cannot display all scene data in real time. A variety of devices have appeared continuously due to the development of hardware; even devices of the same type have different system performances.

The tree is a typical and common plant in natural scenes. When modeling large-scale natural scenes, tree models with abundant details of geometric information are very large and also difficult to render rapidly [1]. The realistic rendering of trees uses large amounts of system resources and rendering time. Currently, 3D graphics acceleration technology has rapidly developed, but it can hardly meet the requirements of rendering complex natural scenes. In the real-time visualization of natural scenes, the challenge of automatically adapting to different requirements and software-hardware environments for generating satisfactory natural scenes has already become a hot topic for 3D interactive games, digital city and virtual forest simulation.

An adaptive visualization system automatically adopts the appropriate condition to present the best visual effect based on its resources, equipment, and scene data. Most adaptive rendering acceleration methods mainly speed up the frame rate while they maintain the quality of images [2]. However, visual effect and rendering speed is a pair of interactional factors. The better the visual effect is, the lower the rendering speed is. On the contrary, if we reduce the rendering time, the visual effect will decrease accordingly. The adaptive visualization of interactive natural scenes should synthetically consider the computing power of the device, rendering time of the scene and the visual effect. The visual effect is properly reduced to ensure that the rendering of natural scenes can be completed within a limited time.

Because of the high complexity and large spatial spans of dynamic natural scenes, it takes a long time to render each frame of a natural scene. This delay leads to the visualization of natural scenes obviously lagging behind the movement of a user’s point of view. The delay in visualization will lead to poor human visual perception of the natural scene. In addition, heterogeneity usually exists in the distribution of trees in a dynamic natural scene. The trees in some places may grow very densely, while the trees in other places may grow relatively sparsely. When the eye moves between the area of dense trees and that of sparse trees, there will be a sudden slowing down or a sudden speed up because of the unbalanced rendering time of the scene. The question of how to automatically adapt to the changes of the complex scenes within a specified time and dynamically adjust simulation information to generate the best natural scene is very important in the visualization of interactive natural scenes.

To automatically adapt to all types of devices that have different software and hardware configurations, this paper proposes a device-independent and time-critical adaptive visualization method for natural scenes. In this method, the best rendering strategy is selected by estimating the rendering time of trees based on their visual importance. To demonstrate the effectiveness of this method, it is applied on different devices, such as a desktop computer, laptop, Pad, and smart phone. Applications show that the method can adapt to devices of different computing powers and system resources, preserve the reality of natural scenes, achieve better visual realism and realize the persistent frame rate when roaming natural scenes.

## Related work

### Adaptive visualization

The mechanism of time-critical computing is that the computing process should be completed before the deadline. The system makes a comprehensive consideration of the resources and computing power currently available, as well as the time and quality requirements of computing. A simple calculation method and reduction of visual quality are adopted when it is necessary to ensure that the computing be completed in a specified time period [3].

To adaptively adjust to image quality while maintaining a stable and constant frame rate, Funkhouser proposed an adaptive algorithm for interactive frame rates in rendering complex virtual scenes through the management of discrete level of details (LODs) [4]. The algorithm performs a constrained optimization to choose a level of detail and rendering algorithm for each potentially visible object to generate the best possible image within the target frame time. Martin described the design and development of an adaptive environment for rendering of 3D models over networks [5]. This environment monitors the distribution of the available resources and selects the appropriate transmission and representation modalities to match these resources. Later Li et al. proposed a time-controlling algorithm for large-scale terrain rendering [6]; it guarantees that each frame is rendered at a preset time and is independent of the terrain or the viewpoint. XF Cao presented a generic solution for remote adaptive streaming and rendering of large terrain [7] that avoids loading irrelevant or redundant data and requests the most important data first. Park et al. proposed an adaptive rendering engine that renders high spatial resolution satellite images for streaming services on the web and manages large-scale satellite image data in current system resources [8]. Bao et al. proposed a framework for rendering large-scale forest scenes realistically and quickly that can automatically extract the level of detail (LOD) tree models [9]. To progressively render virtual scenes from a blurred state to a clear state, sequences of LOD models are transferred from coarse to fine. Reference [10] proposed a novel kd-tree based on a parallel adaptive rendering approach. A two-level framework for adaptive sampling in parallel is introduced to reduce the computation time and to lower the memory usage. In the field of digital city, Zhang applied an adaptive visualization method to the digital city models that can adapt to the changes of virtual scenes in the intended time and achieve good visual effect [11]. Shen et al. proposed an adaptive partitioning of image façade that used a series of horizontal or vertical planes to divide the input point cloud data and automatically extract high-level structure [12]. Scherzer D, et al. introduced the notion and main concepts of TC and described a general approach of image-space reprojection, which facilitates the reuse of shading information by the adjacent frames [13].

Most of the existing adaptive visualization methods are aimed at the stage where data are transmitted from the client’s memory to the display terminal. Usually, the computing power is not the same in different clients; a large amount of data needs to be computed and time needs to be budgeted. The key technologies to address these issues are a gradual rendering mechanism, the compression and management of 3D models, the time-critical rendering algorithm and rendering efficiency optimization strategy.

### Simplified representation for 3D tree models

Irrespective of the type of device that is used, to achieve the reality of scene visualization in real-time, it is necessary to simplify 3D tree models and to reduce the complexity of scenes. Therefore, some researchers have put forward a series of simplification methods for 3D tree models. The basic idea is to gradually reduce the number of meshes and to preserve the appearance of tree models from the original fine tree models. The existing simplification methods of 3D tree models include geometry-based static simplification and view-dependent progressive representation.

The methods of geometry-based static simplification usually adopt pre-generated different LOD models to represent the same object. Compared with the original model, each model retains a certain level of detail. This method selects different resolution models to render in real time based on the viewpoint, stadia and the area size of models in a virtual scene. To simplify the geometric models with a large number of discrete leaves, a foliage simplification algorithm (FSA) was proposed by Remolar [14]. This algorithm iteratively selects two similar leaves to merge into one leaf for the sake of foliage simplification. Xiaopeng Zhang proposed a method for hierarchical union of organs, introducing the botanical knowledge about leaf phyllotaxy and flower anthotaxy (HUO) [15]. Then, Qingqiong et al. adopted a hybrid polygon model to represent leaves according to the morphological characteristics of leaves (broad leaf or conifer). The hybrid polygon/line model can achieve a higher level of geometric compression and can reduce the amount of meshes [16]. Guanbo Bao et al. proposed a new leaf modeling method that uses textures to simplify triangular meshes of leaves [17] so that the visual effect and model complexity can be balanced well; it can render a large scale forest containing thousands of trees in real time. The method of geometry-based static simplification is a good way to reduce the complexity of tree models. However, the simplified model still contains many meshes, and takes a long time to calculate illumination and real-time shadow, which still will be a heavy burden in the rendering process of large scale forest scenes. In a virtual ramble, the different resolution models in a continuously changing scene often occur as hopping. Hopping will affect the human vision perception of natural scenes [18].

The method of view-dependent progressive representation takes the locations between trees and viewpoints in virtual scenes into account and simplifies tree models in real time according to the dynamic viewpoint of a virtual scene. Cook et al. proposed a stochastic simplification of aggregate detail [19]. By using this method, scenes are rendered by randomly selecting a subset of the geometric elements and by altering those elements statistically to preserve the overall appearance of a virtual scene. The amount of simplification depends on a number of factors, including screen size, motion blur, and depth of field. Gumbau presented a view-dependent multi-resolution model for the foliage of the trees [20]. This method interactively reduces the amount of geometric patches that represents the foliage by using a view-dependent multi-resolution scheme. These existing methods of view-dependent progressive representation can solve the hopping problem, but they cannot reflect human vision perception of tree models that can embody topological semantics in a dynamic simulation.

## Methods

### Simplified representation of tree models for different devices

The geometric representation and simplification technology based on LOD can effectively reduce the surface details of models and generate multi-resolution LOD models. However, the simplified model still contains many meshes, and it needs a long time to calculate illumination and real-time shadow. That delay is a heavy burden on the rendering process of a natural scene. However, the image-based rendering method can be used to solve this problem. It renders natural scenes under any viewpoint through the blending of multiple images [21]. It has a great simplification degree because the complexity of models is only related to the number of sampling images, and it has nothing to do with the complexity of original models [22]. However, due to the limitations of image resolution, the models will be fuzzy when observed from a close distance. It is difficult to cope with dynamic changes in light conditions, and the method cannot generate the dynamic effects of natural scenes [23]. Therefore, this paper combines the visual perception features of tree models and adopts a hybrid representation method of geometry and image for different devices to achieve the balance between model simplification and visual quality.

**Fig 1:**
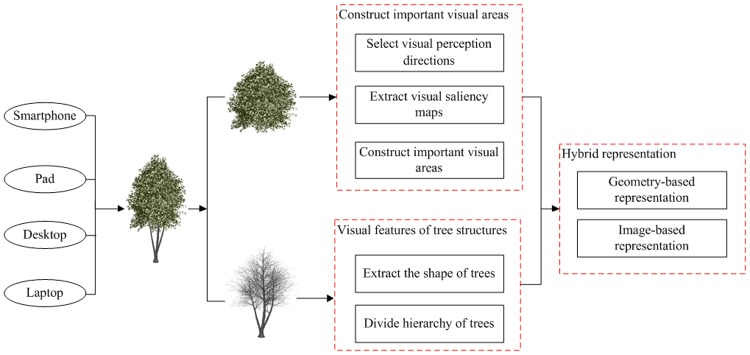
Hybrid representation of a tree model. Tree models are represented using two different parts: topological structure and crown. Only the trunks and main branches of the trees are retained to maintain topological structure, while the branches, twigs and other levels of detail are pruned. As for the simplification of the crown, the saliency map is extracted from the original image for every visual perception direction in turn, and the crown is divided into several important visual areas and unimportant visual areas based on the visual attention model. Furthermore, two different approaches are adopted to represent tree models: geometric representation and image-based representation.

Based on the different performances of all types of devices, this paper uses a hybrid representation method of geometry and image to construct a tree model. This method can achieve the rapid simplification of tree models and effectively compress the data of models, thus reducing the complexity of a natural scene. Because of different organizational structure, texture color and material properties between the trunk and the crown of tree models, trees can be separated into two different parts: the trunk and branch and the leaf. The hybrid representation method of geometry and image for 3D tree models is shown in Fig. 1.

As for the trunk and branches, we divide different hierarchies of tree models based on its unique topological semantics, including trunk, main branches, branches, twigs and so on. To simplify the tree models, we only retain the trunks and main branches of trees, while the branches, twigs and other levels of detail are pruned. As for the crown, based on the visual attention model [24], we select several typical visual perception directions according to the space structure of tree models that form the sequence of the original images used in visual perception. We extract the saliency map from the original image for every visual perception direction in turn and divide the crown into important visual areas and unimportant visual areas.

Finally, based on the partition of the important visual areas for the crown, this paper adopts a heterogeneous simplification method to achieve the geometric simplification of the crown. To preserve the visual perception of tree models and to maintain the important visual leaves with high visual attention, this paper uses the geometric culling method. Textures are used to represent the unimportant visual leaves, greatly reducing the amount of geometric data of unimportant visual leaves and decreasing the complexity of the crown.

However, considering the difference in computing power and system resources between different devices, we adjust the percentage of geometry representation and image representation for the tree models adaptively, according to the graphics hardware performance.

**Fig 2:**
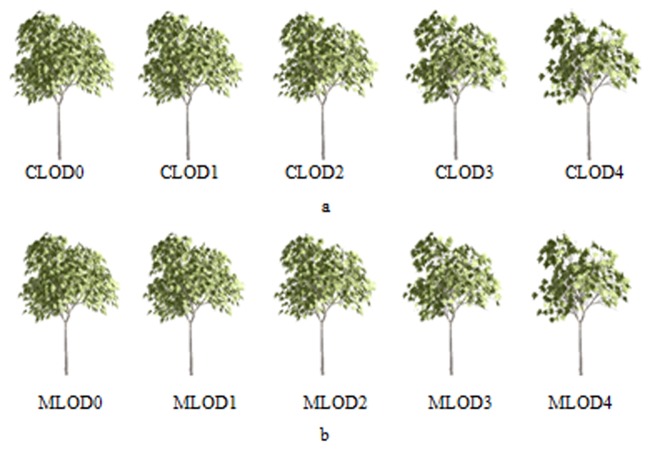
LOD models of Platanus orientalis. a. LOD models for a computer. These models with fine details may consume more rendering time and resources, but they can describe more details of trees. They can be used on computers. b. LOD models for mobile devices. These models are simplified by using LOD technology because mobile devices usually have worse performance than computers.

Fig. 2 displays ten Platanus orientalis models with different levels of detail. The geometric patch numbers of the Platanus orientalis models in Fig. 2a are 6563, 4250, 2137, 925 and 312. However, we use more images to represent the Platanus orientalis models in Fig. 2b, and their geometric patch numbers greatly decrease. The number of geometric patches for Platanus orientalis models in Fig. 2b is 356, 178, 96, 58 and 9. As for the equipment with better graphics hardware configurations, such as desktop computers, we use more meshes to represent tree models, such as the Platanus orientalis models shown in Fig. 2a. As for mobile devices, the proportion of image representation for tree models is higher, and we will adopt the Platanus orientalis models (S1 Dataset) as shown in Fig. 2b.

### Dynamic culling based on visual importance of leaves

The existing tree models usually contain a large amount of geometric detail to represent their elaborate shapes and complex structure. Currently, many 3D graphics acceleration technologies have been proposed, but they can not render complex virtual scenes in real time. Therefore, irrespective of the device used to visualize natural scenes, we must dynamically cull tree models to further improve the rendering efficiency and real-time roaming speed.

This paper uses a geometry culling method for the simplification of important visual leaves to reduce the number of meshes of the crown. For preserving the visual perception of pruned leaves, we adopt a culling equation, as shown in Formula (1), to dynamically prune the leaves [25].

ε(l)=k1∗dis(l,c)+k2∗cos(l,cam)+k3∗area(l) (1)

where *l* is a leaf of tree model, c is the geometric center point of crown, and cam is the visual perception direction. *dis(l*, *c)*, *cos(l*, *cam)*, *area(l)* are the culling factors, where dis *(l*, *c)* represents the distance between the geometric center of leaf and crown, *cos(l*, *cam)* represents the orientation angle between the normal vector of leaf and the visual perception direction, *area(l)* represents the geometric area of leaves. The value of each factor is between 0 and 1, and *k*_*1 +*_ *k*_*2 +*_ *k*_*3*_ = 1. In this paper, the weight values of *k*_*1*,_ *k*_*2*.,_ *k*_*3*_ are 0.3, 0.4 and 0.3, respectively.

In the dynamic culling of leaves based on visual importance, the method first calculates culling factors for each leaf and obtains the value of *ε*(*l)* according to Formula (1). Then, it sorts the important visual leaves in descending order according to the value of *ε*(*l)* and stores the information of sorted leaves into a queue. Finally, based on the distance *d* between the viewpoint and tree models, the rendering rate λ of the important visual leaves is calculated, as shown in Formula (2).

$$\lambda =\frac{1}{Ln\text{(}d)}$$ (2)

where *Ln(d)* is the natural logarithm,*d* is the distance between the viewpoint and the geometric center of tree model. If the number of important visual leaves is *N*, it will dynamically render the first *λN* leaves in the sorted queue. Thus, it can decrease the geometric data of tree models and improve rendering speed [25].

Some leaves are pruned after the above operation, which makes the crown appear sparse and decreases the density of leaves. To preserve the density of leaves and the area similarity of the crown, it is necessary to enlarge the areas of other remaining leaves and to maintain the crown areas. If the number of important visual leaves is *N*, the average area of leaves is *a*, and the sum of area is *S* = *Na* For maintaining the crown areas, if the average area of leaves after pruning is *a*’, the equation *Na = λNa*' is established. Then, the area of leaves after pruning is $a^{'}=\frac{1}{\lambda }a$. According to the comparison of the experimental results [25], if the area of leaves after pruning is $a^{'}=\frac{1}{\sqrt{\lambda }}a$, it will be better than the former. So, the area s' of all leaves after pruning will enlarge to$\frac{1}{\sqrt{\lambda }}$, as shown in Formula (3).

$$s'=\frac{1}{\sqrt{\lambda }}s$$ (3)

where *λ* is the rendering rate, *s*' is the area of all leaves after pruning, and *s* is the area of all leaves before pruning.

The geometry culling method of leaves in this paper calculates the culling factors of important visual leaves and sorts them according to the value of visual perception importance. Then, it dynamically prunes the leaves according to the distance between the viewpoint and the tree models. With the viewpoint observing the trees from near-field to far-field, the number of leaves will decrease, and the area of leaves will gradually enlarge, and vice versa.

### Adaptive visualization of natural scenes

This section mainly discusses how to automatically adapt and generate a satisfactory natural scene on different devices. There are some key technologies, such as model selection, the estimation of rendering time, and the evaluation of object importance.

### Selection of tree models

When rendering the same data on different devices, there will be a difference in rendering speed because of the differences in system performance (such as graphic card, memory, CPU, and operating system). Therefore, the original configuration parameters will no longer be applicable, and they should be reconfigured.

The traditional method of model selection chooses the appropriate models for trees according to the distance to a viewpoint [17]. When the distance to a viewpoint is near, the refined 3D models are used to render. Because of the limit of the visual field, there are not too many trees that are rendered at the same time. Usually, there will be about two or three refined 3D tree models. When the distance to a viewpoint is far away, the simplified geometric LOD models are used. And the farther the tree is to the viewpoint, the rougher the LOD model is. When the distance to a viewpoint is much farther, the visual perception of individual trees is not so obvious, and textures are adopted to represent trees, generally using billboard technology. If there are n + 1 different resolutions of LOD models for each tree, from the highest resolution to the lowest, each model is named as *CLOD0*, *CLOD1…CLODn*. The models used on mobile devices are named as *MLOD0*, *MLOD1…MLODn*. Which model is selected depends on the distance to a viewpoint in a virtual scene, as shown in Formula (4).

$$M=\{\begin{array}{c} CLOD_{0}{}^{}or^{}MLOD_{0}{,}^{}0\leq d\leq d_{1}\\ CLOD_{1}{}^{}or^{}MLOD_{1}{,}^{}d_{1}\leq d\leq d_{2}\\ \vdots \\ CLOD_{n}{}^{}or^{}MLOD_{n}{,}^{{}^{}}d_{n}\leq d \end{array}$$ (4)

Where *0 ≤ d_1_ ≤ d_2_ ≤ … ≤ d_n_* and *d*_*i*_ (*i* = 1, 2, …, *n*) represents the threshold of model selection, and *d* represents the distance from the tree to the viewpoint.

### Estimation of rendering time

To reduce the instability of the rendering time of natural scenes and to make the rendering time to be in a reasonable range of user-specified time, it must estimate the rendering time of a virtual scene.

At present, most of the researches on rendering time estimations of geometric objects are free of textures. Funkhouser [4] presented a linear expression for rendering time of a geometric object that contains a vertex number, polygon number and pixel number. Wimmer and Schmalstieg [26] noted that the expression proposed by Funkhouser only gives the lower limit of the rendering time and that the actual rendering time is related to vertex transformation and rasterization. Therefore, the time estimation function is a linear expression of the polygon vertex number and the pixel number.

With the increase of natural scene complexity, an increasing proportion of texture is used in the visual model of a natural scene. If the resolution of tree texture is much higher, the conversion time and scheduling time in a graphics card will not be ignored. To obtain a more accurate estimation time, the rendering time of a 3D model is expressed as [27]:

$$T=c_{1}\times v\_num+c_{2}\times t\_size$$ (5)

where *v_num* is the polygons of tree model, *t*_*size* is the amount of textures needed to be rendered, *c_1_*and *c_2_* depend on the hardware and software environment of a device and are adaptively adjusted according to the device.

In the process of estimating the rendering time for a natural scene, it first selects n trees of the same level. Then, it computes the amount of polygons, textures and rendering time of the selected n trees. The information of tree *i* is (*T*_*i*,_ *v_num*_i_, *t*_*size*_*i*_). For tree *i*, it can get the rendering time *T_i_* by using the amount of polygons (*v*_*num*_i_), and the amount of textures (*t*_*size*_*i*_), as shown in Formula (6).

$$\{\begin{array}{l} T_{1}=c_{1}\times v\_num_{1}+c_{2}\times t\_size_{1}\\ T_{2}=c_{1}\times v\_num_{2}+c_{2}\times t\_size_{2}\\ \vdots \\ T_{n-1}=c_{1}\times v\_num_{n-1}+c_{2}\times t\_size_{n-1}\\ T_{n}=c_{1}\times v\_num_{n}+c_{2}\times t\_size_{n} \end{array}$$ (6)

Therefore, *n* trees will obtain n equations, and any two equations can obtain a set of *c*_*1*_ and *c*_*2*_. Take *T*_*i*_ and *T*_*j*_ as an example, we can get *c*_*1*_ = (*T*_*i*_ × *t*_*size*_*j*__*T*_*j*_ × *t_size*_*j*_ *)/(t_size*_*j*_ *× v_num*_*i*_ *- t_size × v_num*_*j*_)and. *c*_*2*_ = *(T*_*i*_ × *v_num*_*j -*_ *T*_*j*_ *× v_num*_*i*_ *)/(t_size*_*i*_ *× v_num*_*i*_ *- t_size*_*j*_ *× v_num*_*i*_)

For the applicability of this evaluation method, this paper computes any two equations, and calculates the average values of *c*_*1*_ and *c*_*2*_. The average values of *c*_*1*_ and *c*_*2*_ can be represented as Formula (7) and Formula (8) respectively.

$$c_{1}=\frac{\sum_{\text{i}=1}^{\text{n-1}}\sum_{j=i+1}^{n}\frac{\left(T_{i}\times t\_size_{j}-T_{j}\times t\_size_{i}\right)}{\left(t\_size_{j}\times v\_num_{i}-t\_size_{i}\times v\_num_{j}\right)}}{C_{n}^{2}}$$ (7)

$$c_{2}=\frac{\sum_{\text{i}=1}^{\text{n-1}}\sum_{j=i+1}^{n}\frac{\left(T_{i}\times v\_num_{j}-T_{j}\times v\_num_{i}\right)}{\left(t\_size_{i}\times v\_num_{j}-t\_size_{j}\times v\_num_{i}\right)}}{C_{n}^{2}}$$ (8)

where $C_{n}^{2}$ means the combination of selecting two trees from n trees. When rendering a natural scene, it collects the monitoring data of rendering time. The time estimation function is dynamically adjusted according to the collected monitoring data, and making the time estimation more accurate. The amended *ci* (*i* = 1, 2) is defined in Formula (9).

ci'=(1+β×tact−testtest)×ci (9)

where *t*_*est*_ is the estimated time of all trees in view frustum, *t*_*act*_ is the actual rendering time needed, and *β* is a value between 0 and 1, which reduces the volatility of this feedback algorithm [27].

To evaluate the error between estimated time and actual time [27], suppose the estimated time is *t*_*est* and the actual time is *t*_*act*, then the error between them is defined in Formula (10).

$$error=\frac{\left|t\_act-t\_est\right|}{t\_act}$$ (10)

Table 1 presents the comparison between the estimated time and the actual time when there are 30 trees in view frustum. The testing is conducted on a desktop computer with the following configuration: Intel(R) Core(TM) i3 CPU 550@3.20GHz, 512M graphic card memory, 2G memory. In Table 1, the first column is the five moments sampled when roaming in a natural scene, ET is the estimation time, AT is the actual time, and error is the error between them. The value of error is controlled in a reasonable range, demonstrating that the method of estimating rendering time proposed in this paper is feasible. Table 2 and Table 3 are the comparison of the iPhone and iPad.

**Table 1:** Comparison between estimated time and actual time on computers (unit: s).

Time	ET*(s)*	AT*(s)*	error
t1	0.0695	0.0769	9.6%
t2	0.0657	0.0603	8.9%
t3	0.0616	0.0679	9.3%
t4	0.0687	0.0729	5.7%
t5	0.0636	0.0696	8.6%

**Table 2:** Comparison between estimated time and actual time on the iPhone (unit: s).

Time	ET*(s)*	AT*(s)*	error
t1	0.0598	0.059	1.4%
t2	0.0599	0.066	9.2%
t3	0.0599	0.062	3.4%
t4	0.0600	0.066	9.1%
t5	0.0601	0.065	7.5%

**Table 3:** Comparison between estimated time and actual time on the iPad (unit: s).

Time	ET*(s)*	AT*(s)*	error
t1	0.0626	0.070	10.5%
t2	0.0684	0.075	8.8%
t3	0.0674	0.071	5.1%
t4	0.0660	0.072	8.3%
t5	0.0695	0.075	7.3%

### Evaluation of tree importance

The evaluation of tree importance is a crucial aspect to realize adaptive visualization. When the estimated rendering time exceeds the specified time, it can reduce the LOD levels for relatively unimportant trees to shorten rendering time. When the estimated rendering time is less than the specified time, it can improve the LOD levels for important trees to increase rendering time, thus making the rendering time stable.

**Fig 3:**
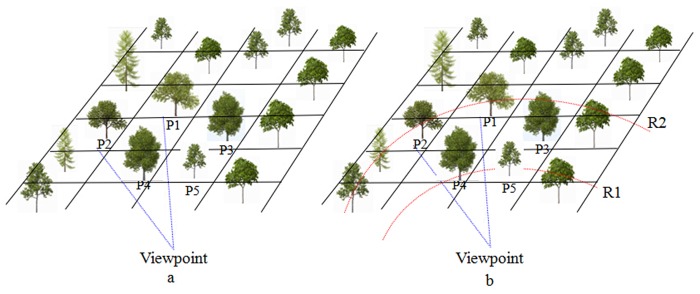
Evaluation of tree importance. a. Our method to evaluate tree importance. The level of detail for a tree is related to viewpoint, tree position, visual angle and tree size. P4 has a higher level of LOD than P3 because P3 has a longer distance to viewpoint. P1 has the same distance to viewpoint as P2, but P1 has a higher level of LOD because it has a smaller visual angle. Although P4 and P5 have the same distance to viewpoint and the same visual angle, P4 has a higher level of LOD than P5 because P4 has a larger crown. b. Comparison with traditional LOD. Using traditional LOD method, P1 and P3 between range R1 and R2 have lower LOD level than P5, which is different from the result of our method.

The tree importance calculation based on the distance is simple and efficient. Trees near the viewpoint have a higher importance, while those far from the viewpoint have a lower importance. However, in actual visual effects, the detail degree of a tree is not only related to its viewpoint location but also related to visual angle. The area in the direct sight is distinct, and the area observed out of the direct sight is relatively vague. As shown in Fig. 3a, there are two trees, P1 and P2, and they have the same distance to the viewpoint. The blue dotted line represents the distance between the tree and the viewpoint, and the sight direction is along VP1. Obviously, the level of detail for tree P1 should be higher than that of tree P2. That is, with the same distance to the viewpoint, the bigger the visual angle is, the less detail it has. Trees near the sight direction have sensitive visual effects and more details.

Compared with traditional LOD, the method proposed in this paper to evaluate the importance of tree can achieve better visual effects. For the traditional LOD, trees in the range of R1 usually have more details than those trees between the range R1 and R2, as shown in Fig. 3b. However, P1 and P3 between range R1 and R2 can be seen more easily than P5 in the range of R1. Therefore, to take the visual angle and size of a tree into consideration is necessary.

This paper uses the projection area of tree crown to measure the size of a tree. As shown in Fig. 3a, there are two trees, P4 and P5, which are assumed to have the same distance to the viewpoint and the same visual angle. Because the tree crown of P4 is larger than that of P5, the visual effect of P4 is obviously better than P5. Therefore, this paper synthetically considers the influences of distance, visual angle and the size of tree crown to express visual effect [27]. A determiner factor related to visual angle is introduced, that is, *factor* = tan*θ*. The formula for the improved measurement of tree importance is as follows:

$$I=\frac{B}{\alpha_{1}\text{(1}+\tan\theta \text{)}\times d+\alpha_{\text{2}}\times \frac{\text{100}}{\text{s}}}$$ (11)

where *α*_*1*_ and *α*_*2*_ are weight coefficients, *B* is the benchmark value of importance, *d* represents the actual distance of a tree from the viewpoint and *s* represents the projection area of tree crown. The value of *B* is calculated from a tree (called a benchmark tree) in the importance-critical area, as shown in Formula (12).

$$B=\alpha_{1}\text{(1}+\text{tan}\theta_{0}\text{)}\times d_{\text{0}}+\alpha_{\text{2}}\times \frac{\text{100}}{s_{0}}$$ (12)

where *θ*_*0*_ is the visual angle of the benchmark tree, *d*_*0*_ is its distance to the viewpoint, and s_0_ is the crown size of the benchmark tree. The larger the value of *I* is, the greater the visual importance is. When the value of *I* is more than 1, it means that the visual effect of this tree is more important, and it is not allowed to reduce its LOD level.

Take tree P1 as the benchmark tree and its importance value as 1; for the marked five trees in Fig. 3a, their importance values are shown in Table 4, where *d* is the distance to viewpoint, *θ* is visual angle, *s* is the projection area of the tree crown, and *I* is the visual importance value.

**Table 4:** Importance information (α1 = 0.6, α2 = 0.4).

Tree position	d	θ	S	I
P1	15	0	6	1
P2	15	30	6	0.75
P3	15	45	9	0.69
P4	9	25	11	1.35
P5	9	25	5	0.98

**Fig 4:**
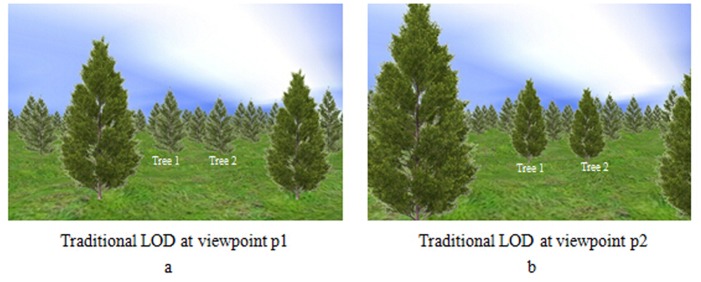
Natural scene of traditional LOD. a. Scene at viewpoint p1 with traditional LOD. With traditional LOD method, at viewpoint p1, the LOD level of Tree 1 and Tree2 is LOD2. b. Scene at viewpoint p2 with traditional LOD. At viewpoint p2, the LOD level of Tree 1 and Tree 2 is LOD1.

**Fig 5:**
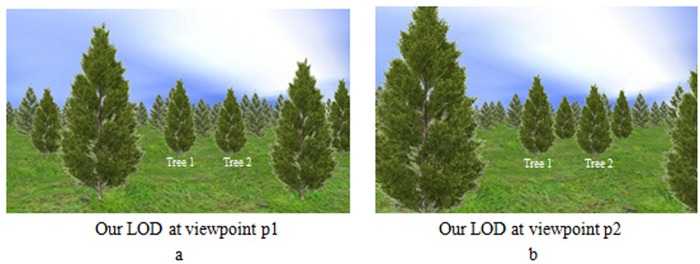
Natural scene of our LOD method. a. Scene at viewpoint p1 with our method. With our method, the LOD level of Tree 1 and Tree 2 is LOD1. b. Scene at viewpoint p2 with our method. At viewpoint p2, the LOD level of Tree 1 and Tree 2 is LOD1.

To verify this evaluation method of tree importance have better visual effects, this paper provides a comparison about the temporal coherence with traditional LOD method. Fig. 4a shows the scene rendered by using traditional LOD method at viewpoint p1, and Fig. 4b shows the same scene after walking forward at viewpoint p2 (S2 Dataset). The scene rendered by using the method proposed in this paper is shown in Fig. 5. Fig. 5a shows the scene at viewpoint p1, and Fig. 5b shows the same scene as Fig. 4b at viewpoint p2. According to these scenes, the LOD level of Tree 1 and Tree2 in Fig. 4a is LOD2, while the LOD level of Tree 1 and Tree2 in Fig. 4b is LOD1. However, the LOD level of Tree 1 and Tree2 is LOD1, no matter in Fig. 5a and Fig. 5b. That means our method can keep better temporal coherence than traditional LOD method, especially for the important objects.

### Time-critical adaptive visualization

**Fig 6:**
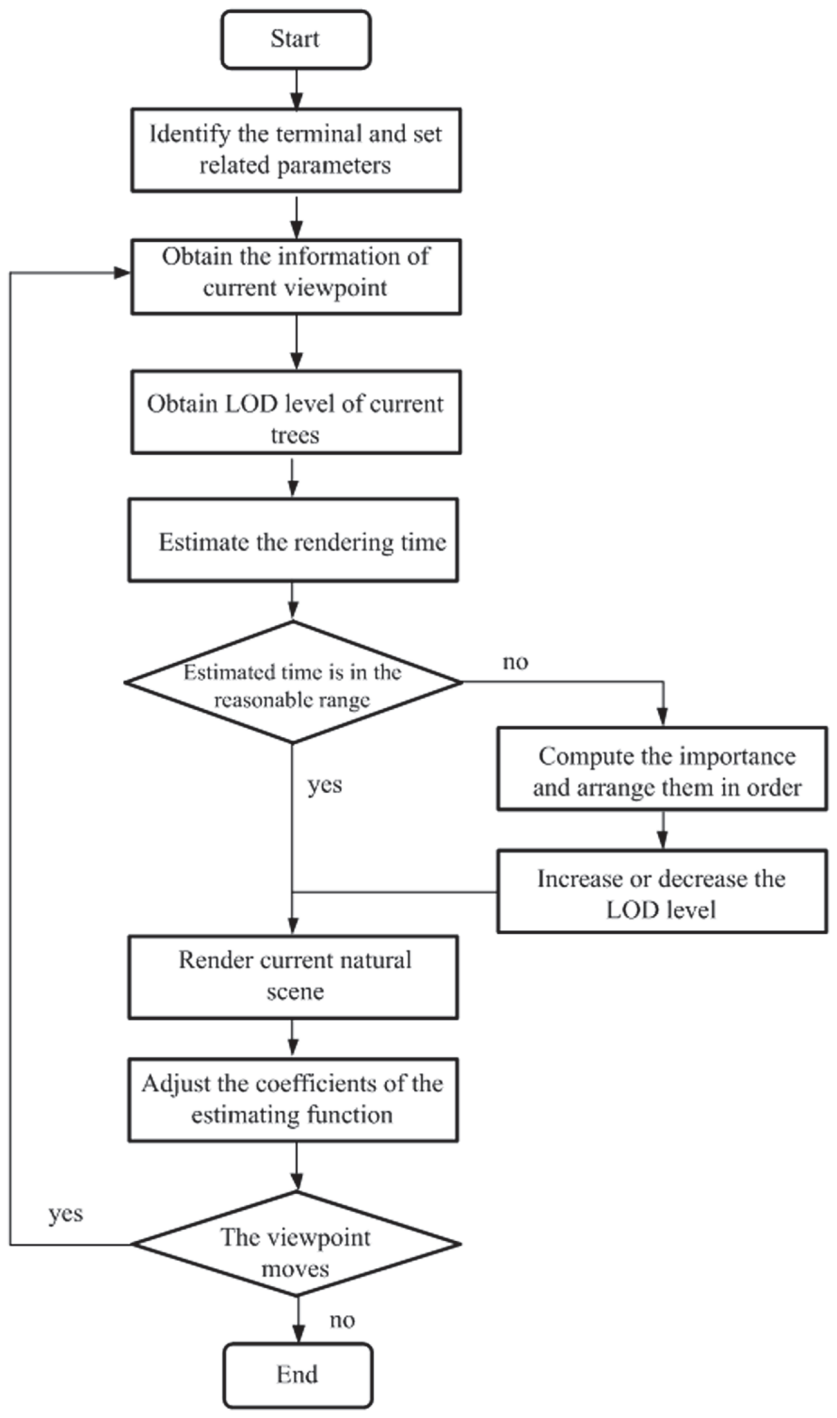
Workflow of time-critical adaptive visualization. This adaptive visualization approach chooses an initial set of tree models with different LODs according to the device type and then estimates the rendering time of the trees in view frustum under the current level of detail. If the estimated rendering time is not in the reasonable range of a specified time, it will adjust the level of LOD for tree models and render natural scenes using the appropriate level of detail.

Because of the specified time, not all tree models will be rendered using the most elaborate LOD model in a natural scene. The proper LOD model is selected for each tree by using the device-independent and time-critical adaptive visualization algorithm. To render natural scenes within a limited time, the model decreases the LOD level of some tree models to reduce rendering time. Certainly we will not decrease the LOD of the trees that have important visual effect [27]. The workflow of the device-independent and time-critical adaptive visualization approach is shown in Fig. 6.

The steps of the adaptive visualization approach are as follows:

Step1: Identify the types of device and set the corresponding parameters for the device.
Step2: Obtain the viewpoint information when the roaming begins, and the LOD level of the trees is obtained by the distance to the viewpoint.
Step3: Estimate the rendering time of the trees in view frustum under the current LOD level.
Step4: Adjust the models of trees. If the estimated rendering time is in the reasonable range of a specified time, the scene is rendered directly. If the estimated time is less than the reasonable range of a specified time, it arranges the trees in the order of importance value from large to small, and increases the LOD level of trees in turns, until all trees are rendered or until all trees reach the highest LOD level. If the estimation time is more than the reasonable range of a specified time, it arranges the trees in the order of importance value from small to large and decreases the LOD level of trees in turns, until the estimated rendering time approximates the specified time.
Step5: Render the natural scene using the appropriate LOD level described in Step 4 and collect the monitoring data of the actual rendering time. The coefficients of the time estimation function are adjusted according to the monitoring data, thus improving the accuracy of the time estimation algorithm.
Step6: Judge whether the viewpoint moves. If the viewpoint moves, then jump to Step 1 and obtain the information of the current viewpoint. If the viewpoint does not move, then the roaming is finished.

## Results and Discussion

### Rendering framework of interactive natural scenes

**Fig 7:**
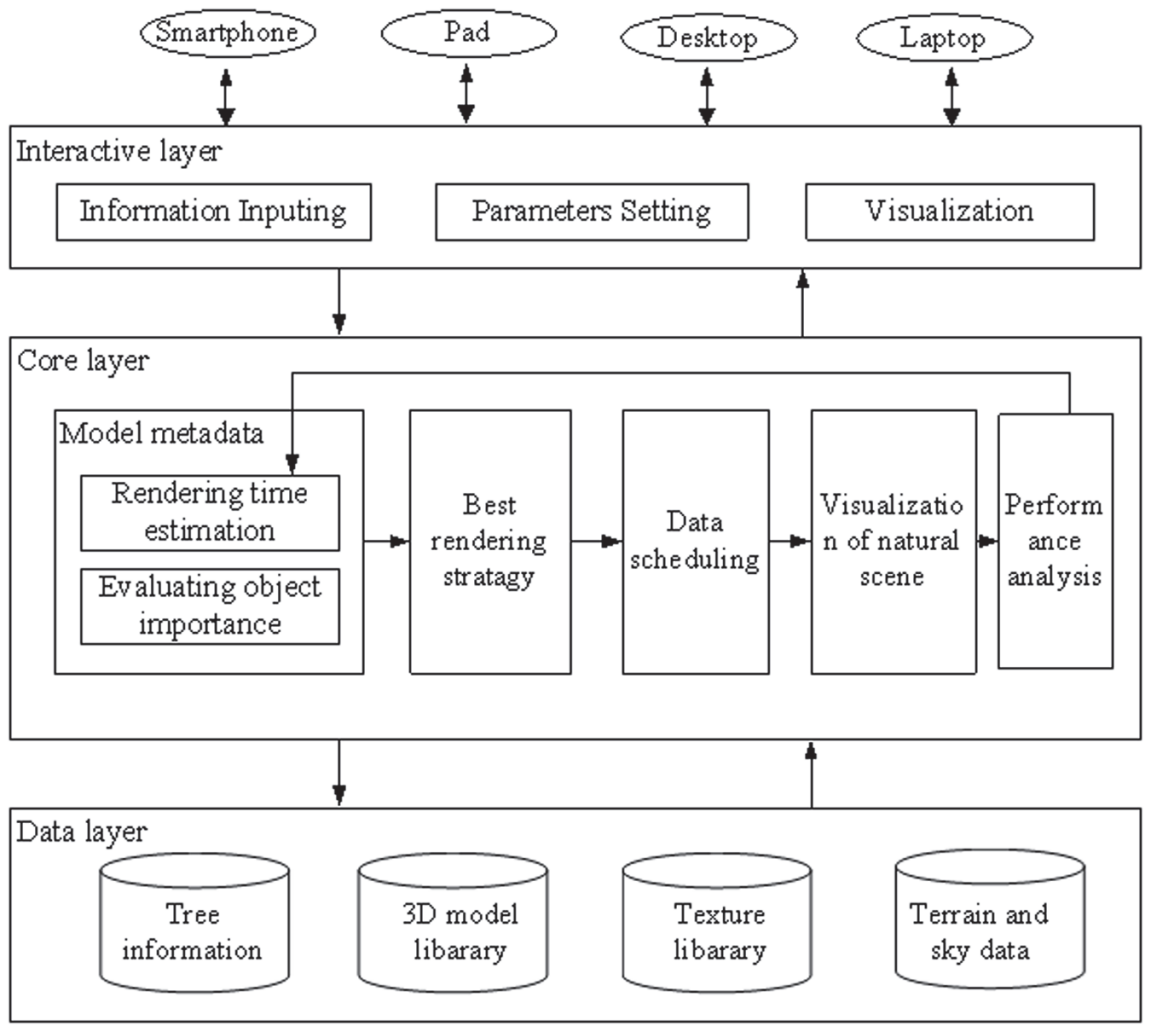
Rendering framework of natural scene. The framework includes three layers: interactive layer, core layer and data layer. In the interactive layer, users set initial data based on their requirements and roam virtual scenes. In the core layer, the system adjusts the rendering strategy according to the data set in the interactive layer and then renders natural scenes. The tree models, textures and other data used in the core layer are all stored in the data layer.

The interactive natural scene simulation system in this paper realizes the adaptive rendering on different devices. This system automatically adapts to different user requirements and device configurations to generate satisfied visual results. The system framework is shown in Fig. 7 and is divided into three levels: user interaction layer, system core layer, and data layer. The application of hierarchical structure can make the system logic level more clear. Different modules interact with each other by transmitting data and messages; this interaction will reduce dependences and coupling relationships.

Interactive layer: this layer provides the interactive functions between the system and the users, including data initialization, display of natural scenes and virtual roaming. Users initialize the parameters for complex natural scenes through this layer, including the number of trees in the scene, planting mode, expected frame rate, etc. After the initialization, it renders the initial natural scene according to these parameters, and users can roam in the natural scene.

Core layer: this layer mainly includes a rendering time estimation module, object importance evaluation module, best rendering strategy module, data scheduling module, visualization module and performance analysis module. The rendering time estimation module is used to estimate the rendering time and to provide the time information to the rendering strategy module. The object importance evaluation module computes the importance of each object for choosing the appropriate level of models. According to the estimated rendering time of each tree model and the results of the object importance evaluation, the best rendering strategy module selects an appropriate model level for each object in the case of total rendering time within the limited time and provides the best visual effect possible. The data scheduling module uses viewpoint parameters to retrieve model data within the current sight ranges from multi-scale databases in real time. The visualization module renders the trees on a 3D terrain based on their settled location after the calculation and realizes the visualization of complex natural scenes in the computer. The performance analysis module collects the monitoring data of every frame and provides an analysis data set for the time evaluation module, subsequently transmitting the analysis results to the rendering time evaluation module.

Data layer: this layer mainly focuses on data access logic (data definition, updating, management, and maintenance, etc). It is used to separate data access and data source and mainly includes the tree information, terrain data, 3D tree model library, texture library, etc. The tree information includes the position, species, heights, ages, biomass and diameter at breast height, etc. A 3D model library stores all tree models with different levels of detail. The texture library includes all types of textures, including that of the sky, landscape and trees.

### Simulation experiment and discussion

**Fig 8:**
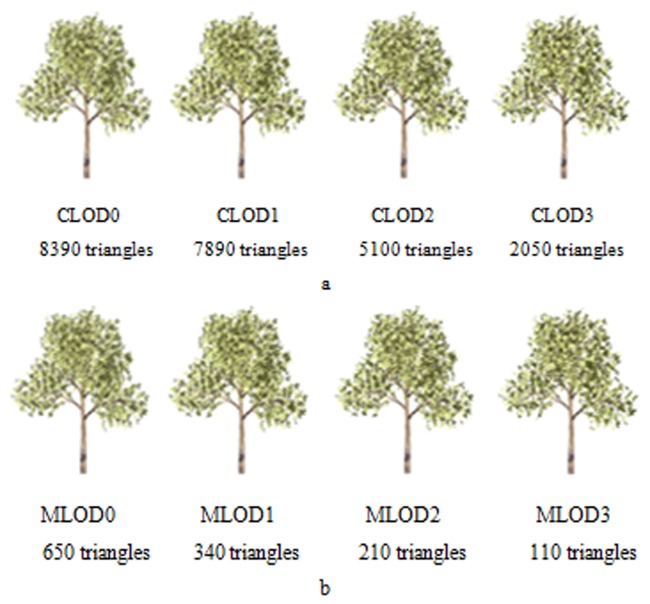
LOD models of Lycium barbarum. a. Tree models with different LODs on a computer. Tree models are represented by triangles, and the number of triangles for each model is shown. The tree model with more details has larger amount of triangles, but it costs more time to render. b. Tree models on mobile device. These models have lower levels of detail than those models used on a computer because mobile devices usually have worse computing power and less system resources.

**Fig 9:**
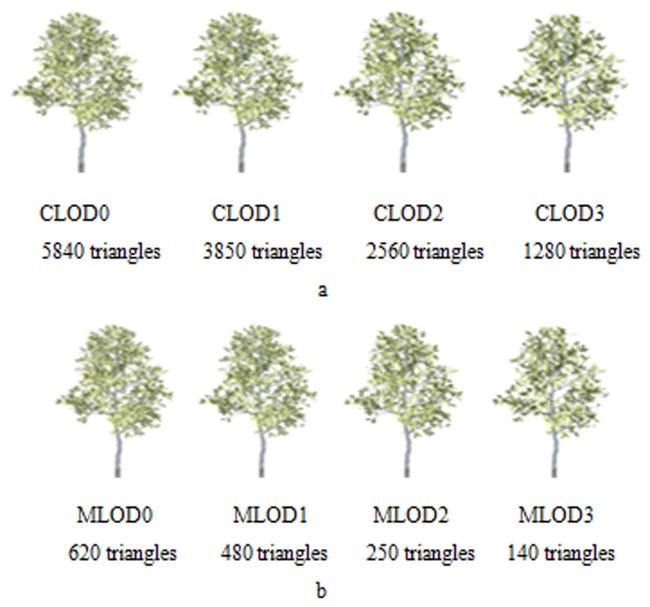
LOD models of Quercus pyrenaic. a. Tree models with different LODs on a computer. b. Tree models on mobile device. This figure shows another type of tree, and the models applied on mobile devices also have a lower level of detail than those models used on a computer.

The tree models for applications on different devices are shown in Fig. 8 and Fig. 9; they are Lycium barbarum and Quercus pyrenaica, respectively. The models in Fig. 8a and Fig. 9a are used on computers, and those in Fig. 8b and Fig. 9b are used on mobile devices, such as the iPhone and the iPad. Each tree has 8 different LODs ordered by the resolution from high to low. The subtitle is the level of LOD and the number of polygons for the models.

We have tested this method on different devices, including computers, the iPhone, and the iPad. To reach a satisfactory roaming rate of 15 fps, that is 0.067 s of the specified time, we compared the rendering time of using or not using the time-critical adaptive visualization approach. The natural scene is planted with Lycium barbarum and Quercus pyrenaic, which are distributed randomly. There are approximately 30~100 trees in the view frustum, and the natural scenes are rendered using these two approaches. The walkthrough of the natural scene is in the same path. The walkthrough distance consists of 500 frames, with every 5 frames as a sample.

Table 5 is the average frame rate before and after using the adaptive visualization approach on different devices. The average frame rate keeps at approximately 15 fps on the devices with different configurations (the configurations are shown in Table 6, Table 7, Table 8 and Table 9). This result demonstrates that the adaptive visualization approach proposed in this paper can dynamically display good visual effects depending on the system resources, the amount of data and so on.

**Table 5:** Average frame rate on different devices.

**Device**	**Before (fps)**	**After (fps)**
Computer 1	10.06	14.93
Computer 2	10.32	14.89
iPhone	11.23	15.38
iPad	9.01	13.89

**Table 6:** Configuration of computer 1.

**Device Name**	**Configuration**
Graphic card	ATI Radeon HD 4550, 512M
Memory	2G (DDR2 SDRAM)
CPU	Intel(R) Core(TM) i3 CPU 550@3.20GHz
Operating system	Windows XP 32bit

**Table 7:** Configuration of computer 2.

**Device Name**	**Configuration**
Graphic card	Nviidia Quadro 600, 1G
Memory	4G (DDR3)
CPU	Intel(R) Xeon(R) CPU E5506 @2.13GHz quad-core
Operating system	Win 7 32bit

**Table 8:** Configuration of iPhone4s.

**Device Name**	**Configuration**
Graphic card	Imagination PowerVR SGX543
Memory	512M(RAM) 16G(ROM)
CPU	Apple A5 1GHz
Operating system	iOS 5.0

**Table 9:** Configuration of iPad4.

**Device Name**	**Configuration**
Graphic card	Imagination PowerVR SGX543MP4
Memory	1G(DDR3) 32G(ROM)
CPU	Apple A6X 1.4GHz
Operating system	iOS 6.0

**Fig 10:**
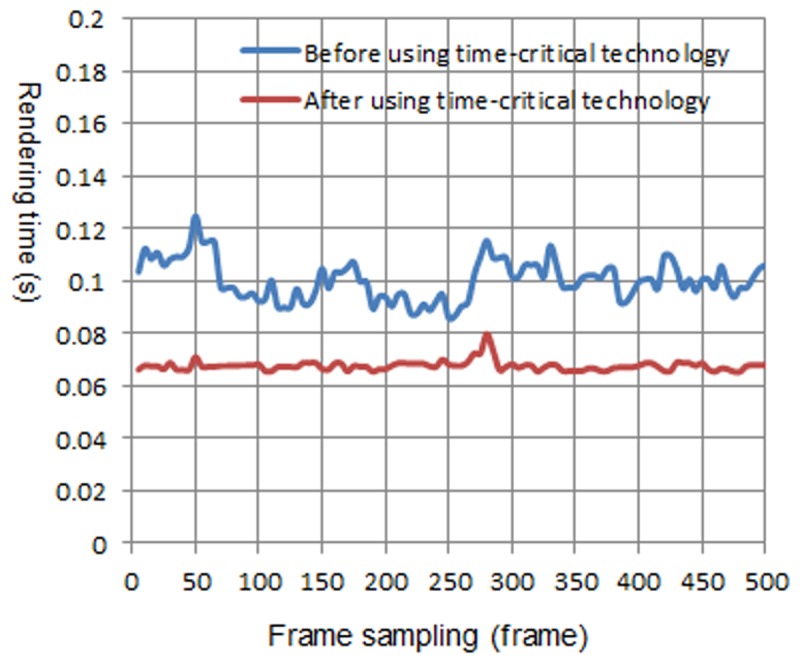
Comparison of rendering time on computer 1. The blue line represents the rendering time before using time-critical technology on computer 1, while the red line represents the rendering time after using time-critical technology. The red line is much smoother, and it takes less time to render natural scenes than the blue line, indicating that time-critical technology is effective and reasonable.

**Fig 11:**
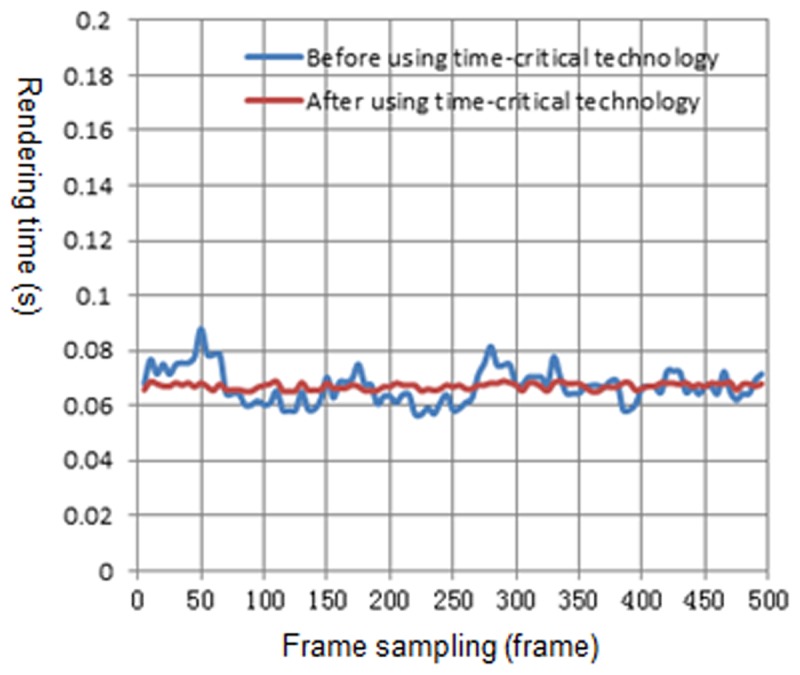
Comparison of rendering time on computer 2. The blue line represents the rendering time before using time-critical technology on computer 2, while the red line represents the rendering time after using time-critical technology. Even if their rendering time is similar, the red line is smoother; indicating that time-critical technology can provide a more stable frame rate.

**Fig 12:**
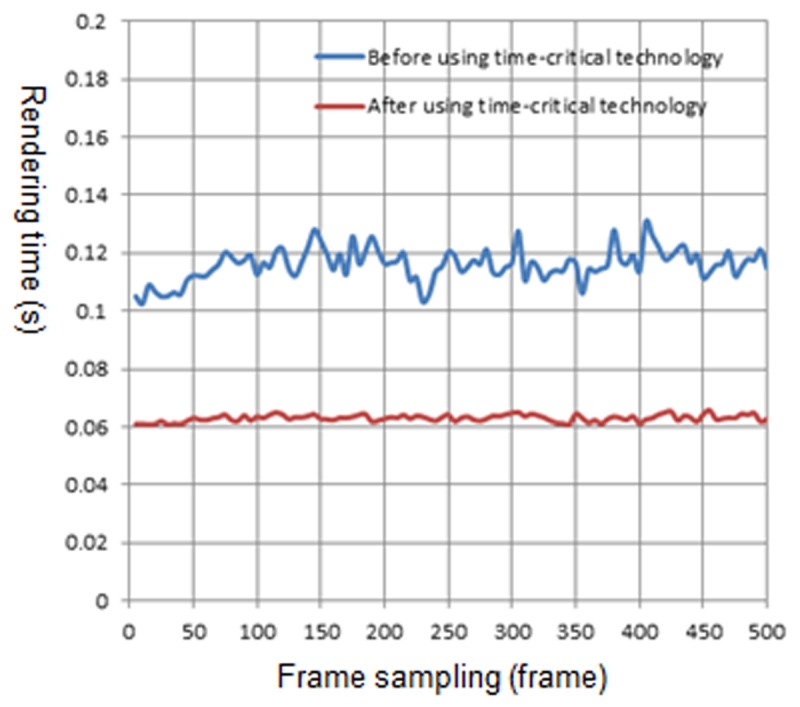
Comparison of rendering time on iPhone. The blue line represents the rendering time before using time-critical technology on iPhone, and it always takes more rendering time than the red line. Furthermore, the red line is also smoother than the blue line; indicating that time-critical technology is effective and reasonable.

**Fig 13:**
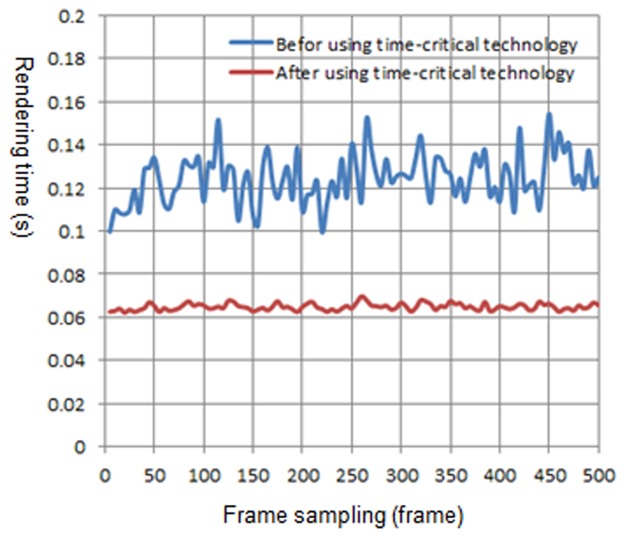
Comparison of rendering time on iPad. The red line representing the rendering time after using time-critical technology is much smoother than the blue line, while it also costs much less rendering time than the blue line.

Fig. 10 is the rendering time before and after using the time-critical adaptive visualization approach on computer 1. There are 30~100 trees in the view frustum. The blue curve represents the rendering time of the natural scene before using the adaptive visualization approach, while the red curve represents the rendering time after using the approach. It can be observed from Fig. 10 that after using the adaptive visualization approach, the rendering time of the natural scene is reduced, and it approximates the specified time. Fig. 11, Fig. 12 and Fig. 13 show the rendering time before and after using the time-critical adaptive visualization approach on computer 2, the iPhone and the iPad, respectively.

Table 10 presents the LOD distribution of trees before and after using this adaptive visualization approach on computer 1. T1~T5 are five different moments, and there are approximately 30 trees in view frustum. Before using the adaptive visualization approach, there are too many LODs of fine details, making the rendering time much longer. After using the adaptive visualization approach, the LODs of fine detail decreased, and the LODs of less detail but with more textures increased. Therefore, the rendering rate of natural scenes is improved by decreasing the LOD level of trees with less visual importance. Table 11, Table 12 and Table 13 present the LOD distribution of trees before and after using this adaptive visualization approach on computer 2, the iPhone and the iPad.

**Table 10:** LOD distributions on computer 1.

	**Before**				**After**			
Time	**clod0**	**clod1**	**clod2**	**clod3**	**clod0**	**clod1**	**clod2**	**clod3**
*T1*	2	3	9	17	1	2	5	23
*T2*	1	2	11	16	1	2	5	23
*T3*	1	4	10	16	0	2	6	24
*T4*	2	4	9	15	1	3	4	23
*T5*	2	3	10	18	1	3	2	24

**Table 11:** LOD distributions on computer 2.

	**Before**				**After**			
Time	**clod0**	**clod1**	**clod2**	**clod3**	**clod0**	**clod1**	**clod2**	**clod3**
*T1*	1	3	11	17	1	3	10	16
*T2*	2	4	9	15	2	5	9	17
*T3*	2	3	12	14	1	4	11	15
*T4*	1	2	12	15	2	2	13	13
*T5*	2	4	8	16	1	5	9	17

**Table 12:** LOD distributions on the iPhone.

	**Before**				**After**			
Time	**clod0**	**clod1**	**clod2**	**clod3**	**clod0**	**clod1**	**clod2**	**clod3**
*T1*	0	10	12	8	1	8	0	21
*T2*	2	8	16	4	2	6	1	21
*T3*	2	10	14	4	2	6	1	21
*T4*	2	10	16	2	2	4	4	20
*T5*	4	8	18	0	4	2	3	21

**Table 13:** LOD distributions on the iPad.

	**Before**				**After**			
Time	**clod0**	**clod1**	**clod2**	**clod3**	**clod0**	**clod1**	**clod2**	**clod3**
*T1*	0	10	14	6	0	3	1	26
*T2*	2	8	14	16	2	0	1	27
*T3*	2	10	14	4	2	0	1	21
*T4*	4	8	14	4	2	0	1	27
*T5*	2	8	8	12	2	1	0	15

Because of the better performance of computer 2 compared with computer 1, computer 2 reaches the average frame rate 16.14 fps before using the adaptive visualization approach. As shown in Fig. 11, the application in computer 2 has unstable fluctuations before using the adaptive visualization approach. After using the time-critical adaptive visualization approach, the frame rate is much more stable. Comparing Table 10 with Table 11, the number of high level LODs on computer 2 is greater than on computer 1. This result is because the computing power of computer2 is better than that of computer 1. Computer 2 decreases the rendering rate by increasing the number of more elaborate models, which keeps the frame rate at the specified value.

Table 14 is the frame rate of the adaptive visualization approach for different tree numbers (30, 50, and 80) tested on computer 1. When there are 30 trees in view frustum, the average frame rate is approximately 15 fps. When there are 50 or 80 trees in view frustum, the average frame rates are correspondingly improved, but they cannot reach 15 fps. There are two important factors that affect the frame rate. One factor is the importance threshold, which may not permit decreasing the LOD level of the trees for the essential visual effect; the other factor is the rendering time of tree models with the least detail. The rendering time of natural scenes must be equal to or greater than the value of the tree number multiplied by the rendering time of the least detailed model. Table 15, Table 16 and Table 17 display the frame rates of adaptive visualization, which is tested on computer 2, the iPhone and the iPad.

**Table 14:** Frame rates for different tree numbers on computer 1.

**Number of trees**	**Before (fps)**	**After (fps)**	**Improvement**
30	10.06	14.93	48.4%
50	6.70	11.22	67.5%
80	4.55	7.90	73.6%

**Table 15:** Frame rates for different tree numbers on computer 2.

**Number of trees**	**Before (fps)**	**After (fps)**	**Improvement**
30	10.32	14.89	44.3%
50	6.85	11.37	66%
80	4.63	7.94	71.5%

**Table 16:** Frame rates for different tree numbers on the iPhone.

**Number of trees**	**Before (fps)**	**After (fps)**	**Improvement**
30	11.23	15.38	37.0%
50	8.33	10.87	30.5%
80	5.0	7.14	42.8%

**Table 17:** Frame rates for different tree numbers on the iPad.

**Number of trees**	**Before (fps)**	**After (fps)**	**Improvement**
30	9.01	13.89	54.1%
50	6.14	9.8	59.6%
80	4.55	7.04	54.7%

**Fig 14:**
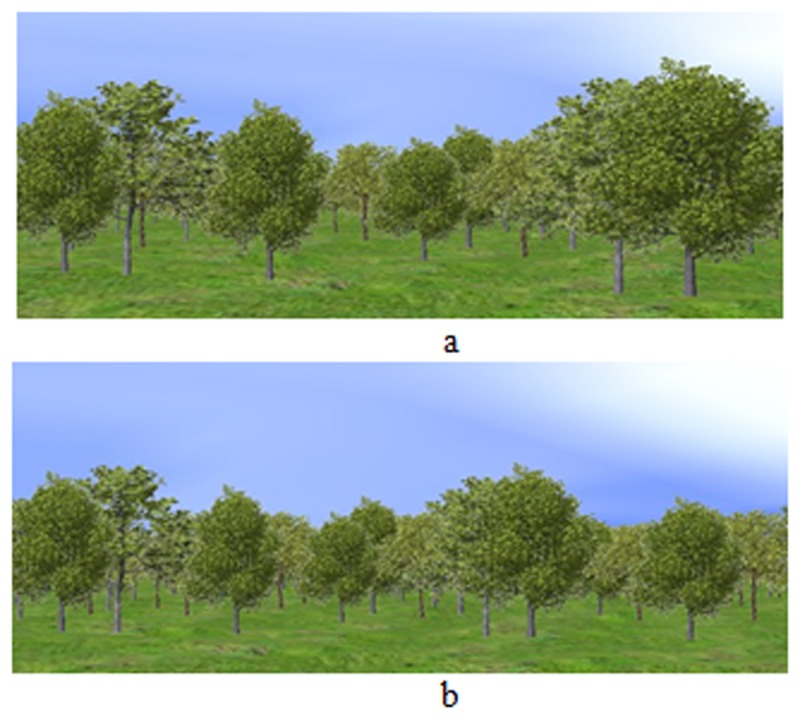
Natural scenes on computers. a. Adaptive visualization approach on computer 1. b. Adaptive visualization approach on computer 2. These two nature scenes are rendered using the adaptive visualization approach presented in this paper. The applications show that the adaptive visualization approach can improve the quality and speed of rendering effectively.

Fig. 14 (S3 Dataset) displays the natural scenes at the moment T1 of Table 10 and Table 11. Fig. 14a is the nature scene using the adaptive visualization approach on computer 1. There are 30 trees in view frustum. Fig. 14b is the nature scene using the adaptive visualization approach on computer 2.

**Fig 15:**
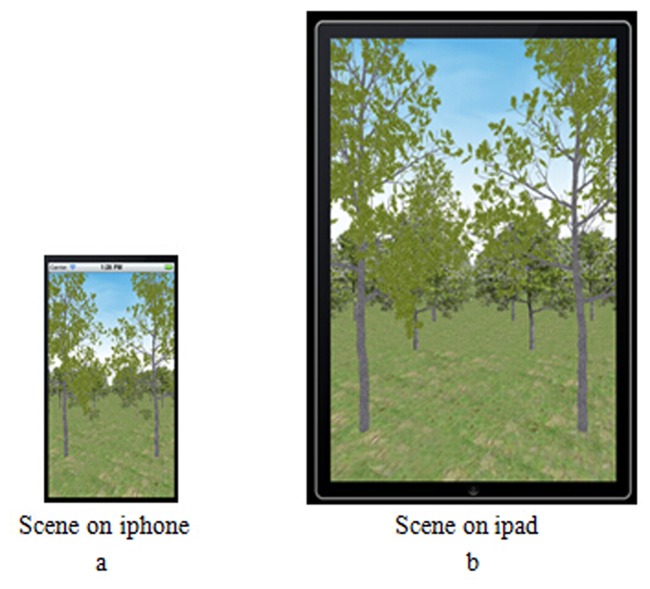
Natural scenes on mobile devices. a. Natural scenes on the iPhone. b. Natural scenes on the iPad. These two nature scenes on mobile devices are also rendered using the adaptive visualization approach presented in this paper. The applications show that the adaptive visualization approach is suitable for mobile devices, and it can improve the quality and speed of rendering effectively.

Additionally, this time-critical adaptive approach for visualizing natural scenes is applied on both iPhone and iPad, as shown in Fig. 15 (S1 Dataset and S3 Dataset). The natural scenes can also be rendered at a constant frame rate.

## Conclusion

This paper describes a hybrid representation method of 3D tree models that are suitable for different devices. The geometry-based and image-based rendering method is used to simplify a 3D tree model to reach a balance between model simplification and visual quality. Then, an approach based on the device-independent and time-critical adaptive generation of interactive natural scenes is proposed. By estimating rendering time and computing tree importance, the appropriate LOD tree models are selected to render in natural scenes. The rendering time is controlled in a reasonable range of a specified time. Moreover, the speed of eye movements is an essential factor influencing object importance in a natural scene. The fast moving trees in natural scenes can only be observed in a short time, leading to the vague visual effects. The speed of eye moving should be taken into account in future research because it is closely related to visual importance.

In addition to the application in natural scene rendering, the time-critical adaptive approach proposed in this paper can be applied in some related fields, such as augmented reality and mobile visual search. Many augmented reality systems are designed with vision-based wide-area registration algorithm, and dynamically enhance the real scenes with objects data stored on devices [28–31]. Taking an augmented city guide or navigational tool as an example, people use location-based service on mobile devices to enhance their experience. The augmented reality system should fully consider the features of mobile devices, including limited computing abilities, battery capacity, and user’s response time. Therefore, the time-critical adaptive approach can be extended to render the augmented reality scenes and make GPS positioning; thus the augmented reality system can provide better user experience.

Mobile visual search or mobile visual location recognition [31] is a kind of location-based service, which can be applied in many fields of our lives, such as hygiene, outdoor object search, entertainment, etc. By using the mobile visual location recognition applications, the users can get more information about the objects with its image of the landmark. And the system can do the recognition operations just using the query image of landmark. The existing mobile location recognition applications commonly use the image retrieval method to do the location recognition operations. For example, mobile landmark search [32–35] extracts compressed visual descriptors on mobile devices, which only consider the visual and location information. At the same time, these mobile retrieval methods [32–33] usually use image retrieval and compressed descriptors to identify the location. The compressed descriptors [36–38], such as tags, image of landmark, are also based on visual and local feature information. These applications can adopt an extended time-critical adaptive approach to get better user experience, such as taking the viewpoint, distance or size into consideration. What’s more, these applications applied on different devices also can consider the different capabilities of mobile devices, which are taken into account in this paper. For example, compression ratio of image can be decided by the devices. The devices with better computing abilities can have a relatively low compression ratio. Therefore, a suitable mobile visual search strategy can be adopted for different devices.

## Supporting Information

S1 Dataset Data of tree model. (ZIP)

S2 Dataset Data of tree model. (ZIP)

S3 Dataset Data of tree model. (ZIP)
